# A Novel Voltammetric Approach for the Rapid Quantification of Rosmarinic Acid in *Origanum micranthum* and *Origanum minutiflorum*

**DOI:** 10.1002/cbdv.71894

**Published:** 2026-11-01

**Authors:** Elif Mollahasanoğlu, Gökçe Öztürk, Tuğba Subaş, Ufuk Özgen, Merve Badem, Şeyda Kanbolat, Dilek Kul, Fatma Ağın, Esen Sezen Karaoğlan, Sıla Özlem Şener, Sevda Türk, İnci Selin Doğan

**Affiliations:** ^1^ Faculty of Pharmacy, Department of Analytical Chemistry Karadeniz Technical University Trabzon Türkiye; ^2^ Department of Analytical Chemistry, Graduate School of Health Sciences Karadeniz Technical University Trabzon Türkiye; ^3^ Faculty of Pharmacy, Department of Pharmacognosy Karadeniz Technical University Trabzon Türkiye; ^4^ Faculty of Pharmacy, Department of Biochemistry Karadeniz Technical University Trabzon Türkiye; ^5^ Faculty of Pharmacy, Department of Pharmaceutical Botany Atatürk University Erzurum Türkiye; ^6^ Gulhane Faculty of Pharmacy, Department of Pharmacognosy University of Health Sciences Ankara Türkiye; ^7^ Faculty of Pharmacy, Department of Pharmaceutical Chemistry Karadeniz Technical University Trabzon Türkiye

**Keywords:** boron‐doped diamond electrode, *Origanum*, rosmarinic acid, UV‒vis spectroscopy, voltammetry

## Abstract

In this study, rosmarinic acid (RA) was isolated and quantitatively determined in extracts obtained from *Origanum micranthum* and *Origanum minutiflorum* using complementary analytical techniques. Following the isolation of RA from the ethyl acetate subextract of *O. micranthum*, spectrophotometric analyses were performed to evaluate its distribution in different solvent fractions, revealing a pronounced enrichment in ethyl acetate extracts of both species. In addition, an electrochemical strategy based on differential pulse voltammetry (DPV) was established for the rapid quantification of RA employing a boron‐doped diamond (BDD) electrode. Under optimized experimental conditions, the method exhibited linearity over the concentration range of 0.2–80 µM with a detection limit of 0.061 µM. The applicability of the proposed approach was validated by successful analysis of plant extracts, yielding recoveries of 100.62% for *O. micranthum* and 100.74% for *O. minutiflorum*. The combined use of isolation, spectroscopic evaluation, and electrochemical quantification provides an efficient analytical framework for the determination of RA in the investigated *Origanum* extracts.

## Graphical abstract

A sensitive voltammetric method using a boron‐doped diamond (BDD) electrode enables the quantification of rosmarinic acid (RA) in *Origanum* extracts. UV–Vis spectroscopy reveals higher RA content in ethyl acetate subextracts, while the combined spectroscopic and electrochemical approach provides an efficient strategy for RA analysis in the investigated *Origanum* extracts.

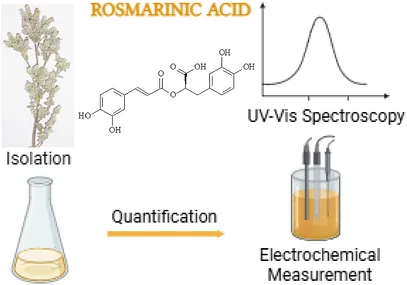

## 1 Introduction

RA is a naturally occurring phenolic metabolite that is widely distributed among plant species belonging to the Boraginaceae family and the Nepetoideae subfamily of Lamiaceae, notably including medicinal plants such as *Rosmarinus officinalis*, *Salvia officinalis*, and *Melissa officinalis* [1]. Extensive pharmacological investigations have demonstrated that RA exhibits a diverse range of biological activities, including strong antioxidant, anti‐inflammatory, anticancer, and antimicrobial properties, which underline its importance as a biologically active constituent in both traditional and modern therapeutic systems [2, 3, 4, 5]. In traditional medicine, plants rich in RA have long been employed in the treatment of respiratory and inflammatory disorders such as asthma, allergic rhinitis, and otitis media, as well as conditions associated with fatigue, weakness, and depressive symptoms [6, 7]. Moreover, clinical studies have provided evidence supporting the therapeutic effectiveness of RA‐containing formulations [8].

The wide spectrum of biological activities associated with RA has led to its increasing incorporation into products ranging from dietary supplements and cosmetics to pharmaceutical preparations [2]. Consequently, the development of analytical approaches capable of providing accurate, rapid, and reliable determination of RA has become essential for both quality control and pharmacological investigations. A variety of instrumental techniques, including spectrophotometric, voltammetric, and chromatographic methods, have been employed for the identification, quantification, and purification of RA. Among these, high‐performance liquid chromatography (HPLC) and gas chromatography–mass spectrometry (GC–MS) are the most commonly used methods for RA analysis in plant materials and food matrices [9, 10, 11, 12]. Despite their high analytical performance, these techniques often involve relatively long analysis times, high operational costs, extensive sample preparation, and the generation of considerable solvent waste, which may restrict their practical applicability. In this context, electrochemical techniques have attracted growing interest as alternative tools for the determination of RA, owing to their inherent advantages such as rapid response, high sensitivity, and potential for miniaturization and on‐site analysis [13]. Previous electrochemical studies on RA determination have employed a variety of carbon‐based electrodes and voltammetric techniques, frequently in combination with different electrode modification strategies. Reported approaches include square‐wave voltammetry using a laccase/1‐n‐butyl‐3‐methylimidazolium hexafluorophosphate‐modified carbon paste electrode [14] and a peroxidase/gold nanoparticle‐ionic liquid in a crosslinked chitosan‐modified carbon paste electrode [15]; DPV using a magnetic molecularly imprinted polymer‐modified carbon paste electrode [16], an ionic liquid/carbon nanotube paste electrode [17], a vertically ordered mesoporous silica film‐modified pre‐activated glassy carbon electrode [18], an Fe‐based metal–organic framework‐modified glassy carbon electrode [19], and a disposable pencil graphite electrode [20]; square‐wave stripping voltammetry using a chitosan/carbon nanotube composite carbon paste electrode covered with DNA [21] and a pretreated glassy carbon electrode [22]; and cyclic voltammetry (CV) using a peptide‐modified graphene oxide screen‐printed carbon electrode [23] and a glassy carbon electrode [24]. The reported analytical performance varies considerably depending on the electrode configuration and voltammetric technique, with LOD values ranging from approximately 0.0018 to 12 µM and substantial variation in the corresponding linear ranges. In the present study, the unmodified BDD electrode combined with DPV provided a linear range of 0.2–80 µM and a LOD of 0.061 µM without requiring additional surface modification. BDD electrodes, in particular, have been widely adopted in electrochemical applications due to their exceptionally low background current, broad potential window, and strong resistance to surface fouling [25]. As a natural phenolic compound containing multiple hydroxyl groups, RA exhibits redox properties that are ideal for electrochemical analysis. The voltammetric analysis of RA using a BDD electrode has not been previously reported and is presented for the first time in this study.

Accordingly, the present study aimed to isolate RA from *Origanum micranthum* Vogel (Lamiaceae), spectroscopically analyze the extracts of *O. micranthum* and *Origanum minutiflorum* O. Schwarz & P. H. Davis with respect to RA content, and develop a sensitive and rapid electrochemical method for the quantitative determination of RA in these extracts.

## 2 Results and Discussion

### 2.1 Isolation Studies

The isolated compound obtained from ethyl acetate subextract of *O. micranthum* (OMC‐E) was structurally characterized by ^1^H‐NMR, ^13^C‐NMR and MS analyses (Figures S1–S3) and identified as RA [26]. The elucidated structure is illustrated in Figure 1, while the corresponding spectral data are provided in the Supplementary Material. The obtained spectroscopic data were in good agreement with previously reported values for RA [27, 28], confirming the successful isolation of the target compound. Although RA has been previously reported in several *Origanum* species including *O. micranthum* and *O. minutiflorum* [27, 28], its isolation in the present study served as a critical step for subsequent spectrophotometric and electrochemical investigations.

**FIGURE 1:**
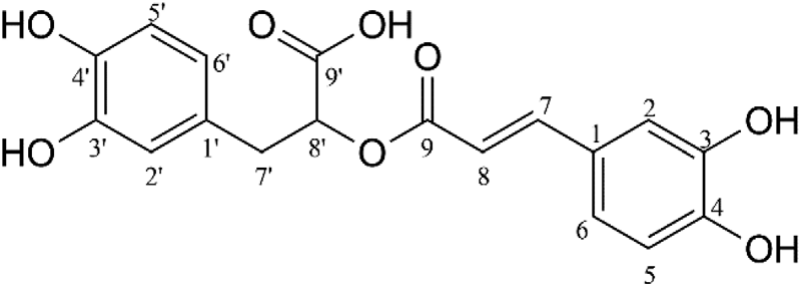
The chemical structure of RA.

**RA**: (C_18_H_16_O_8_, molecular weight: m/z [M + H]^+^ = 361.67); ^1^H‐NMR (400 MHz, CD_3_OD): δ 7.45 (*d*, 1H, H‐7, *J* = 15.9 Hz), 6.95 (*d*, 1H, H‐2, *J =* 1.8 Hz), 6.86 (*dd*, 1H, H‐6, *J* = 8.2 Hz, *J =* 1.8 Hz), 6.68 (*d*, 1H, H‐5, *J* = 8.2 Hz), 6.66 (*d*, 1H, *J* = 1.8 Hz, H‐2′), 6.60 (*d*, 1H, H‐5′, *J* = 8.0 Hz), 6.52 (*dd*, 1H, H‐6′, *J* = 8.0 Hz, *J =* 1.8 Hz), 6.17 (*d*, 1H, H‐8, *J* = 15.9 Hz), 5.09 (*dd*, 1H, H‐8′, *J* = 8.3 Hz, *J =* 4.1 Hz), 3.01 (*dd*, 1H, H‐7′a, *J* = 14.3 Hz, *J =* 3.9 Hz), 2.91 (*dd*, 1H, H‐7′b, *J* = 14.2 Hz, *J* = 8.5 Hz); ^13^C‐NMR (100 MHz, CD_3_OD): δ 174.0 (C‐9′), 168.7 (C‐9), 149.8 (C‐4), 147.8 (C‐3), 147.0 (C‐7), 146.3 (C‐3′), 145.4 (C‐4′), 129.6 (C‐1′), 127.8 (C‐1), 123.3 (C‐6), 121.9 (C‐6′), 117.7 (C‐2′), 116.6 (C‐5), 116.4 (C‐5′), 115.4 (C‐2), 114.7 (C‐8), 75.1 (C‐8′), 38.1 (C‐7′)

### 2.2 Spectrophotometric Studies

Quantitative spectrophotometric analysis was employed to determine the RA content in extracts obtained from *O. micranthum* and *O. minutiflorum*. A calibration curve was constructed using purified RA isolated in this study, demonstrating excellent linearity between absorbance and concentration (*R* ^2^ = 0.999), with absorbance described by the linear regression *A* = 0.0026C (µM) − 0.007. The RA contents of the extracts, expressed as RA equivalents (RAE, µM), are summarized in Table 1. The results clearly demonstrate that OMC‐E and ethyl acetate subextract of *O. minutiflorum* (OMN‐E) contained substantially higher amounts of RA compared to other extracts. This observation can be attributed to the intermediate polarity of ethyl acetate, which favors the extraction of phenolic compounds such as RA. The comparative distribution of RA across different extracts is illustrated in Figure 2, further supporting the preferential enrichment of RA in ethyl acetate fractions.

**TABLE 1:** RA content of the extracts from *O. micranthum* and *O. minutiflorum*.

Extract	RA content (µM) ± standard deviation
OMC‐M^a^	538.333 ± 6.079
OMC‐E^b^	829.615 ± 3.903
OMC‐A^c^	379.359 ± 1.105
OMN‐M^d^	755.000 ± 1.601
OMN‐E^e^	834.359 ± 6.041
OMN‐A^f^	623.077 ± 3.525

*Note*: Values are expressed as mean ± standard deviation (*n* = 3).

^a^Methanol extract of *O. micranthum*.

^b^Ethyl acetate subextract of *O. micranthum*.

^c^Remaining aqueous subextract of *O. micranthum*.

^d^Methanol extract of *O. minutiflorum*.

^e^Ethyl acetate subextract of *O. minutiflorum*.

^f^Remaining aqueous subextract of *O. minutiflorum*.

**FIGURE 2:**
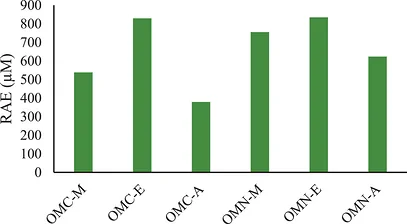
Comparison of RA content of the extracts from *O. micranthum* and *O. minutiflorum*.

### 2.3 Voltammetric Studies

#### 2.3.1 Effect of pH

The electrochemical response of RA is highly dependent on the pH of the supporting electrolyte, as its redox process involves proton‐coupled electron transfer associated with phenolic hydroxyl groups. To systematically evaluate this effect, CV and DPV measurements were performed over a wide pH range using different buffer systems. Representative voltammograms obtained at selected pH values are presented in Figure 3.

**FIGURE 3:**
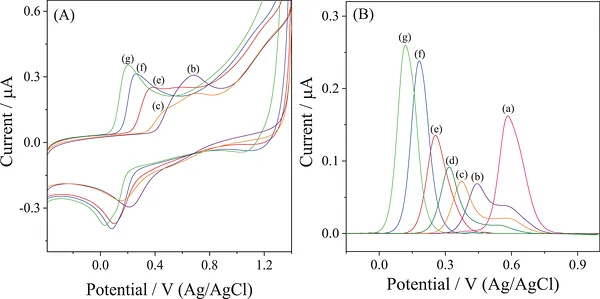
Voltammograms of 40 µM RA obtained by (A) CV in (b) pH 3.0 BRB, (c) pH 4.0 BRB, (e) pH 6.0 PBS, (f) pH 7.0 PBS, and (g) pH 8.0 PBS, and by (B) DPV in (a) pH 0.7 H_2_SO_4_, (b) pH 3.0 BRB, (c) pH 4.0 BRB, (d) pH 5.0 BRB, (e) pH 6.0 PBS, (f) pH 7.0 PBS, and (g) pH 8.0 PBS. Scan rate: 100 mV s^‒1^.

CV measurements were conducted within the potential window of –0.3 to +1.40 V at a scan rate of 100 mV s^−1^ over a pH range of 0.7–10.0. The voltammograms obtained for 40 µM RA displayed a well‐defined anodic peak accompanied by a corresponding cathodic peak in the reverse scan, indicating that RA exhibits both anodic and cathodic responses at the BDD electrode.

In DPV experiments, RA produced distinct anodic peak responses across the investigated pH range, further confirming its electrochemical activity. As the quantitative analysis was based on anodic current responses, all subsequent determinations were performed using the anodic peak currents obtained from CV and DPV measurements.

Among the tested media, PBS at pH 8.0 produced the highest and most symmetrical oxidation peaks, reflecting enhanced electron‐transfer kinetics under mildly alkaline conditions. The dependence of the anodic peak current on pH was further examined by plotting peak current values against pH over the range 0.7–10.0 (Figure 4). These results consistently demonstrated that PBS at pH 8.0 yielded superior signal intensity and peak symmetry for RA. Accordingly, pH 8.0 PBS was selected as the optimal supporting electrolyte for the voltammetric determination of RA at the BDD electrode.

**FIGURE 4:**
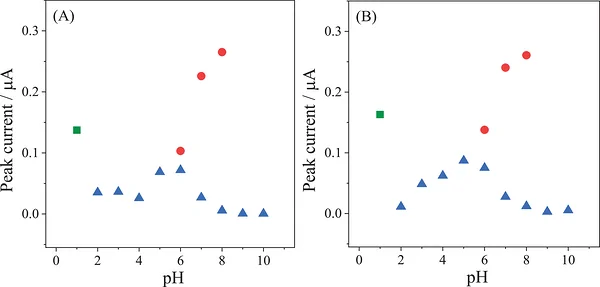
Effect of pH on the peak current of RA for (A) CV and (B) DPV methods. RA concentration: 40 µM. (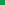) H_2_SO_4_, (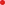) PBS, and (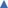) BRB.

The effect of pH on the anodic peak potential (*E*_p_) of RA was further investigated using both CV and DPV to elucidate the role of protons in the electrochemical oxidation process. The results revealed a linear relationship between the *E*_p_ and the solution pH in the range of 0.7 to 8.0. The *E*_p_ of RA shifted toward less positive values with increasing pH, consistent with a proton‐coupled electron transfer mechanism.

The linear regression equations obtained from the CV and DPV measurements are given below, respectively:

(1) $$E_{p}\;\left(\text{mV}\right)=742.15-68.1\;\text{pH}\;\left(R^{2}=0.996\right)$$

(2) $$E_{p}\;\left(\text{mV}\right)=630.96-63.6\;\text{pH}\;\left(R^{2}=0.999\right)$$

The negative slopes of –68.1 mV pH^−1^ obtained from CV and –63.6 mV pH^−1^ obtained from DPV indicate that the electrochemical oxidation of RA at the BDD electrode is governed by a proton‐coupled electron transfer process. According to the Nernst equation, slope values close to the theoretical value of approximately –59 mV pH^−1^ are characteristic of redox reactions involving equal numbers of electrons and protons [29].

#### 2.3.2 Effect of Scan Rate

Scan rate investigations were conducted to clarify the nature of the charge‐transfer process governing the electrochemical oxidation of RA at the BDD electrode. Cyclic voltammetric responses of 8 µM RA in pH 8.0 PBS were recorded at scan rates between 5 and 250 mV s^−^^1^. As shown in Figure 5, a systematic increase in anodic peak current (*I*_p_) with increasing scan rate was observed. A linear dependence of the *I*_p_ on the square root of the scan rate was obtained, which is characteristic of a diffusion‐controlled electrochemical process. This relationship can be expressed by the following equation:

(3) $$I_{p}\;\left(\mu A\right)=0.0101\;v^{1/2}\;\left(\mathrm{mV}\;s^{-1}\right)+0.0185\;\left(\;R^{2}=0.997\right)$$

**FIGURE 5:**
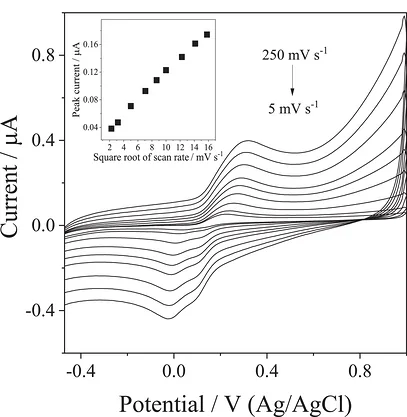
Cyclic voltammograms of 8 µM RA in pH 8.0 PBS at scan rates between 250 and 5 mV s^‒1^. Inset: plot of peak current versus square root of scan rate.

The slope of the plot of log *I*_p_ versus logarithm of the scan rate (log *v*) was determined to be 0.395, which is in close agreement with the theoretical value of 0.5 expected for diffusion‐controlled redox processes. The corresponding regression equation is given by:

(4) $$\log I_{p}\;\left(\mu A\right)=0.395\;\log v\;\left(\mathrm{mV}\;s^{-1}\right)-1.704\;\left(\;R^{2}=0.999\right)$$

These results clearly demonstrate that the electrochemical response of RA at the BDD electrode is governed predominantly by diffusion, with negligible contribution from surface adsorption, thereby supporting the suitability of this system for reliable quantitative analysis.

#### 2.3.3 Determination of RA

To enable the quantitative determination of RA, a calibration study was first carried out under the optimized experimental conditions using DPV method. Working solutions were prepared by appropriate dilution of the RA stock solution with the selected supporting electrolyte. As shown in Figure 6, the anodic peak current increased systematically with increasing RA concentration, indicating a well‐defined analytical response.

**FIGURE 6:**
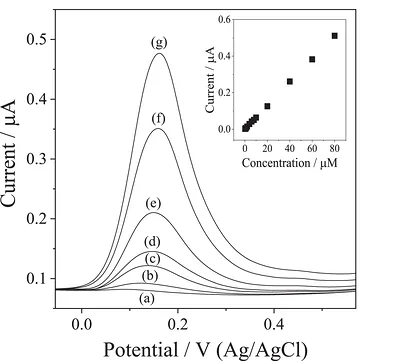
Differential pulse voltammograms of (a) 0.4, (b) 2, (c) 6, (d) 10, (e) 20, (f) 40, and (g) 60 µM RA in pH 8.0 PBS. Inset: Calibration plot of peak current versus RA concentration.

A linear calibration relationship was obtained over the concentration range of 0.2–80 µM, demonstrating that the voltammetric response within this interval is governed by diffusion‐controlled mass transport. The main analytical characteristics of the calibration plot, including slope, intercept, and correlation coefficient, are summarized in Table 2.

**TABLE 2:** Calibration data of RA in pH 8.0 PBS on the BDD electrode by DPV.

Parameter	DPV method
Peak potential (mV)	161
Linearity range (µM)	0.2–80
Slope (µA µM^‒1^)	6.38 × 10^‒3^ ± 2.25 × 10^‒5^
Intercept (µA)	1.05 × 10^‒3^ ± 6.63 × 10^‒4^
Correlation coefficient, *R* ^2^	0.999
LOD (µM)	0.061
LOQ (µM)	0.184
Intraday %RSD of peak potential^a^	0.30
Interday %RSD of peak potential^a^	0.76
Intraday %RSD of peak current^a^	0.83
Interday %RSD of peak current^a^	1.22

^a^Number of experiments: 5, RA concentration: 40 µM.

The sensitivity of the proposed method was evaluated by calculating the limit of detection (LOD) and the limit of quantification (LOQ) according to the expressions LOD = 3*s*/*m* and LOQ = 10*s*/*m*, where *s* denotes the standard deviation of peak currents obtained from three replicate measurements and *m* is the slope of the corresponding calibration curve [30]. The resulting LOD and LOQ values, also given in Table 2, confirm the high sensitivity of the DPV‐based method for RA determination.

The precision of the method was assessed in terms of both intraday and interday variability. Intraday precision was evaluated from five successive measurements of freshly prepared solutions within the same day, while interday precision was determined over two different days. As summarized in Table 2, the relative standard deviation (%RSD) values for both peak current and peak potential remained below 2%, indicating excellent precision and demonstrating the robustness of the proposed voltammetric procedure.

#### 2.3.4 Recovery Studies of RA

To verify the accuracy and applicability of the developed voltammetric method for real sample analysis, recovery experiments were performed using extracts of *O. micranthum* and *O. minutiflorum*. OMC‐E and OMN‐E were prepared in pH 8.0 PBS as described in the experimental section, and their RA contents were subsequently analyzed. The corresponding results are summarized in Table 3.

**TABLE 3:** Recovery data obtained from the solutions of OMC‐E and OMN‐E by DPV method using BDD electrode.

Parameter	OMC‐E	OMN‐E
Added pure RA concentration (µM)	20.000	20.000
Found pure RA concentration (µM)^a^	20.124	20.148
Average recovery (%)	100.62	100.74
%RSD of recovery	1.27	0.95
Bias (%)	0.62	0.74

^a^Number of experiments: 5.

Quantification of RA in the extract solutions was carried out using the regression equation obtained from the calibration study. Recovery values were determined by spiking the extract samples with known concentrations of RA standard and comparing the measured total RA concentrations with the expected values. As shown in Table 3, recoveries of 100.62% for OMC‐E and 100.74% for OMN‐E were achieved.

The high recovery percentages, together with the low %RSD values, indicate acceptable analytical performance in the investigated extracts. These results support the accurate and reliable quantification of RA in the *O. micranthum* and *O. minutiflorum* extracts analyzed in this study.

## 3 Conclusions

In this work, RA was successfully isolated from the ethyl acetate subextract of *O. micranthum* and quantitatively determined in extracts of both *O. micranthum* and *O. minutiflorum*. Spectrophotometric analysis based on Fe^2+^–RA complex formation revealed that the ethyl acetate fractions were markedly enriched in RA compared to the corresponding methanol and aqueous extracts, confirming the suitability of this solvent system for selective RA extraction. In parallel, a sensitive and rapid electrochemical method was developed using a BDD electrode, enabling reliable voltammetric determination of RA in extracts of *O. micranthum* and *O. minutiflorum*. The excellent linearity over a wide concentration range, the low detection limit, and the high recovery values obtained from real samples demonstrate the accuracy, precision, and robustness of the proposed approach. Overall, the integration of chromatographic isolation, spectrophotometric screening, and electrochemical quantification provides an efficient analytical approach for RA determination and demonstrates its potential for further applications in phytochemical analysis.

Notably, the use of a BDD electrode offers distinct advantages for the electrochemical analysis of polyphenolic compounds such as RA, owing to its wide potential window, low background current, and strong resistance to surface fouling. These properties contribute to signal stability and reproducibility, supporting the suitability of the proposed DPV‐based method for RA determination in the investigated *Origanum* extracts.

## 4 Experimental Section

### 4.1 Plant Materials

The aerial parts of *O. micranthum* and *O. minutiflorum* were used as plant material in this study. *O. minutiflorum* samples were collected from Etler Plateau (Antalya, Türkiye; 1800 m) in September 2010, while *O. micranthum* samples were obtained from Kozan (Adana, Türkiye; 1300 m) in August 2009. Botanical identification was performed by qualified taxonomists, and voucher specimens were deposited at the Ankara University Faculty of Pharmacy Herbarium under accession numbers AEF 25873 and AEF 25949, respectively.

### 4.2 Extraction, Fractionation, and Isolation

Vanillin (Fluka), ethyl acetate, methanol, sulfuric acid (Riedel‐de Haën), n‐hexane, and chloroform (Merck) were used in the extraction and isolation studies. Sefadex LH‐20 and silica gel (Kieselgel 60, 0.040–0.063 mm, Merck 9385 and Kieselgel 60, 0.063–0.200 mm, Merck 7734) were used for open column chromatography, and silica gel 60 F254 (20 × 20, Merck 5554) was used for thin layer chromatography (TLC). 1H‐ and ^13^C‐NMR spectra were recorded using a Bruker Avance III 400 MHz spectrometer.

The dried and powdered aerial parts (250 g) of *O. micranthum* were extracted with methanol (1.5 L × 3) at room temperature and filtered. The combined filtrates were condensed to dryness in an evaporator at 40°C and the methanol extract (45 g) was suspended in a mixture of H_2_O:MeOH (9:1). It was then partitioned with chloroform (300 mL × 2) and ethyl acetate (300 mL × 2), respectively. The chloroform, ethyl acetate, and remaining aqueous phases were evaporated to obtain 12, 6, and 24 g subextracts, respectively. The same procedure was also performed for *O. minutiflorum* (50 g), resulting in methanol extract (13 g), chloroform (4 g), ethyl acetate (0.5 g), and remaining aqueous (7 g) subextracts.

The isolation studies were carried out on *O. micranthum*. The OMC‐E (5.5 g) was applied to a silica gel column (Kiesel gel 60, 0.040–0.063 mm Merck, 9385) and gradient elution (80:20:2, 70:30:3, 50:50:5) was performed using CHCl_3_:MeOH:H_2_O solvent system. According to the TLC profile, fractions 27 and 28 were combined and applied to a Sephadex LH‐20 column and eluted with methanol. Fractions 7–9 were combined and RA was obtained.

TLC plates were heated at 110°C for 1–2 min after spraying with 1% vanillin/H_2_SO_4_. ^1^H‐NMR, ^13^C‐NMR, and MS methods were used to elucidate the structure of RA.

### 4.3 Spectrophotometric Studies

CH_3_COONa, CH_3_COOH, and FeSO_4_.7H_2_O were purchased from Sigma‐Aldrich. Pure RA isolated from *O. micranthum* was used as the standard substance. RA solutions were prepared at concentrations of 500, 250, 125, 62.5, 31.25, and 15.625 µM with 0.1 M sodium acetate buffer at pH 6.0. The dry extracts obtained from the aerial parts of *O. minutiflorum* and *O. micranthum* were prepared in the buffer solution to achieve a concentration of 10 mg mL^−1^. In the test tubes, 2000 µL of test solutions were pipetted into the sample blank tube and 2000 µL of buffer was pipetted into the reagent blank tube. FeSO_4_.7H_2_O was added to the test tubes and reagent blank tube to reach a final concentration of 1 mM, and the tubes were vortexed. All tubes were incubated in the dark at 25°C for 1 h. After incubation, the absorbance of the samples was measured at 572 nm using a UV‒vis spectrophotometer (Shimadzu, UV‒1280). The experiments were conducted in triplicate [31].

### 4.4 Voltammetric Studies

For the electrochemical analysis of RA, a 1.0 × 10^−3^ M RA stock solution was prepared by weighing 0.0036 g of RA and dissolving it in ethanol. The solution was then sonicated in an ultrasonic bath for 15 min.

A 0.2 M phosphate buffer (PBS) was prepared at pHs of 6.0, 7.0, and 8.0 using Na_2_HPO_4_ and NaH_2_PO_4_ (Sigma‐Aldrich). A 0.04 M Britton‐Robinson buffer (BRB) was prepared in the pH range of 2.0 to 10.0 using 0.04 M CH_3_COOH (Merck), 0.04 M H_3_PO_4_ (Merck), and 0.04 M H_3_BO_3_ (Sigma‐Aldrich). Additionally, a 0.2 M H_2_SO_4_ (Merck) solution was prepared with a pH of 0.7. All solutions were prepared at room temperature.

All voltammetric measurements were performed using an Autolab analyser, Netherlands. A classical three‐electrode cell was used. A BDD electrode of inner diameter 3 mm and boron doping level of 1000 ppm (Windsor Scientific Ltd.) was polished using alumina slurry on a BASi polishing pad. Pt wire and Ag/AgCl electrodes were used as auxiliary and reference electrodes, respectively. The pH measurements were made on a HANNA pH meter HI2211 equipped with a combined glass pH‐sensor with a pH accuracy of ± 0.05. Ultrapure water was obtained from Sartorius Arium ProUV (resistivity ≥ 18 MΩ cm). All measurements were made at room temperature.

Before each measurement, the working electrode was polished with aluminum hydroxide on a polishing pad. Subsequently, the electrode surface was cleaned by performing four CV scans in a potential range of –0.3 to +1.4 V at a scan rate of 100 mV s^−1^ in a cell containing buffer solution without RA.

A total of 0.0052 g of OMC‐E or OMN‐E were weighed, transferred into volumetric flasks, and made up to 10 mL with ethanol. The solutions were then sonicated in an ultrasonic bath for 15 min. From the prepared stock solutions, 300 µL of *O. micranthum* and 100 µL of *O. minutiflorum* were taken, and the volumes were adjusted to 5 mL with PBS. The differential pulse voltammograms of the solutions were obtained, and the current values were applied to the respective calibration equations to calculate the concentrations of the extract stock solutions. For the recovery study, a 20 µM pure RA was added to the 40 µM extract solution to achieve a total RA concentration of 60 µM.

### 4.5 Statistical Analysis

No transformation, normalization, or formal outlier exclusion was applied to the experimental data. Spectrophotometric results are presented as mean ± standard deviation from three replicate measurements (*n* = 3). Precision and recovery studies were performed using five replicate measurements (*n* = 5), and variability was expressed as %RSD. Calibration data were evaluated by linear regression analysis. No inferential statistical comparisons between experimental groups were performed; therefore, *p* values and post‐hoc analyses were not applicable. Statistical calculations were performed using Microsoft Excel.

## Author Contributions

**Elif Mollahasanoğlu**: writing – original draft, investigation, data curation, validation. **Gökçe Öztürk**: writing – original draft, investigation, data curation, methodology, validation. **Tuğba Subaş**: writing – review and editing, investigation, data curation. **Ufuk Özgen**: conceptualization, project administration, funding acquisition, writing – review and editing. **Merve Badem**: investigation, methodology, data curation. **Şeyda Kanbolat**: investigation, methodology, data curation. **Dilek Kul**: conceptualization, validation, visualization. **Fatma Ağın**: supervision, conceptualization, validation, visualization. **Esen Sezen Karaoğlan**: resources. **Sıla Özlem Şener**: methodology. **Sevda Türk**: validation, visualization. **İnci Selin Doğan**: validation, visualization.

## Conflicts of Interest

The authors declare no conflicts of interest.

## Supporting information



Supporting information for this article is available.
**Supporting File 1**: cbdv71894‐sup‐0001‐SuppMat.pdf

## Data Availability

The data that support the findings of this study are available from the corresponding author upon reasonable request.
