# Supplementary material for: A Novel Voltammetric Approach for the Rapid Quantification of Rosmarinic Acid in Origanum micranthum and Origanum minutiflorum
Source: Chem Biodivers. 2026 Nov 1;23(11):e71894. doi: 10.1002/cbdv.71894 (PMC13724176; doi:10.1002/cbdv.71894)
Supplement: Supplementary file 1 [file CBDV-23-e71894-s001.pdf]

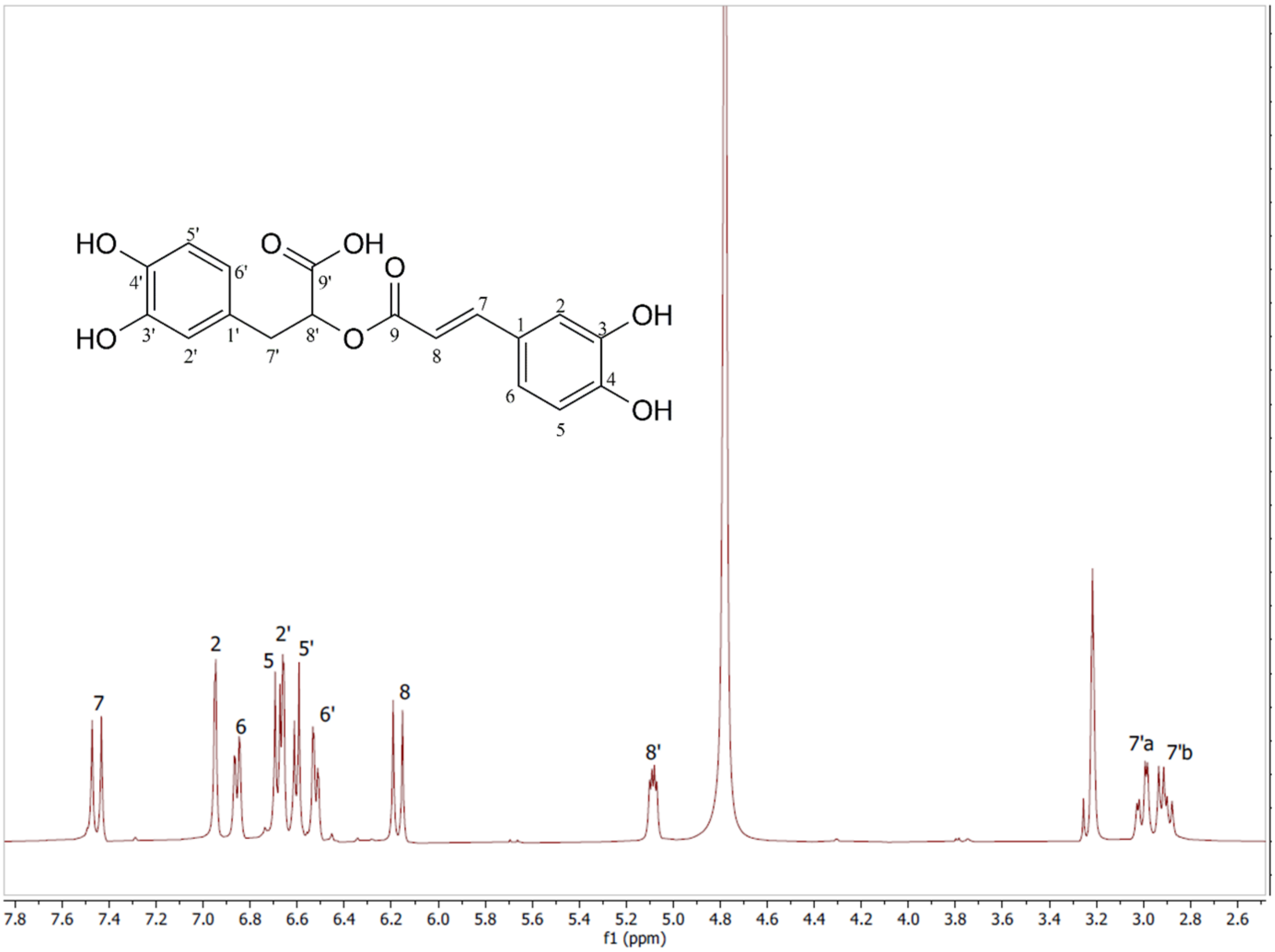

Figure S1. ^{1}H-NMR spectrum of RA (400 MHz, CD_3OD).

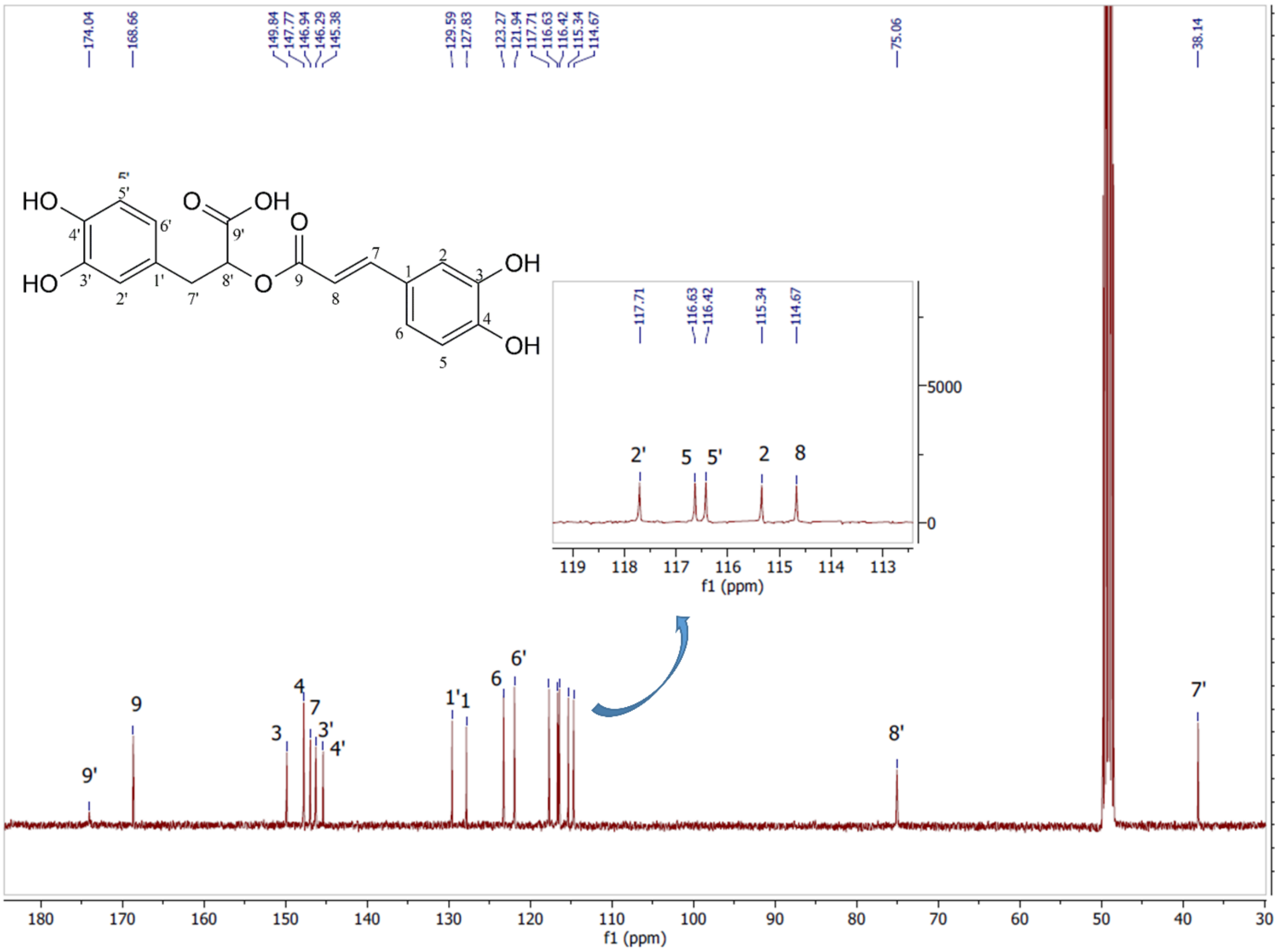

Figure S2. ^{13}C-NMR spectrum of RA (100 MHz, CD_3OD).

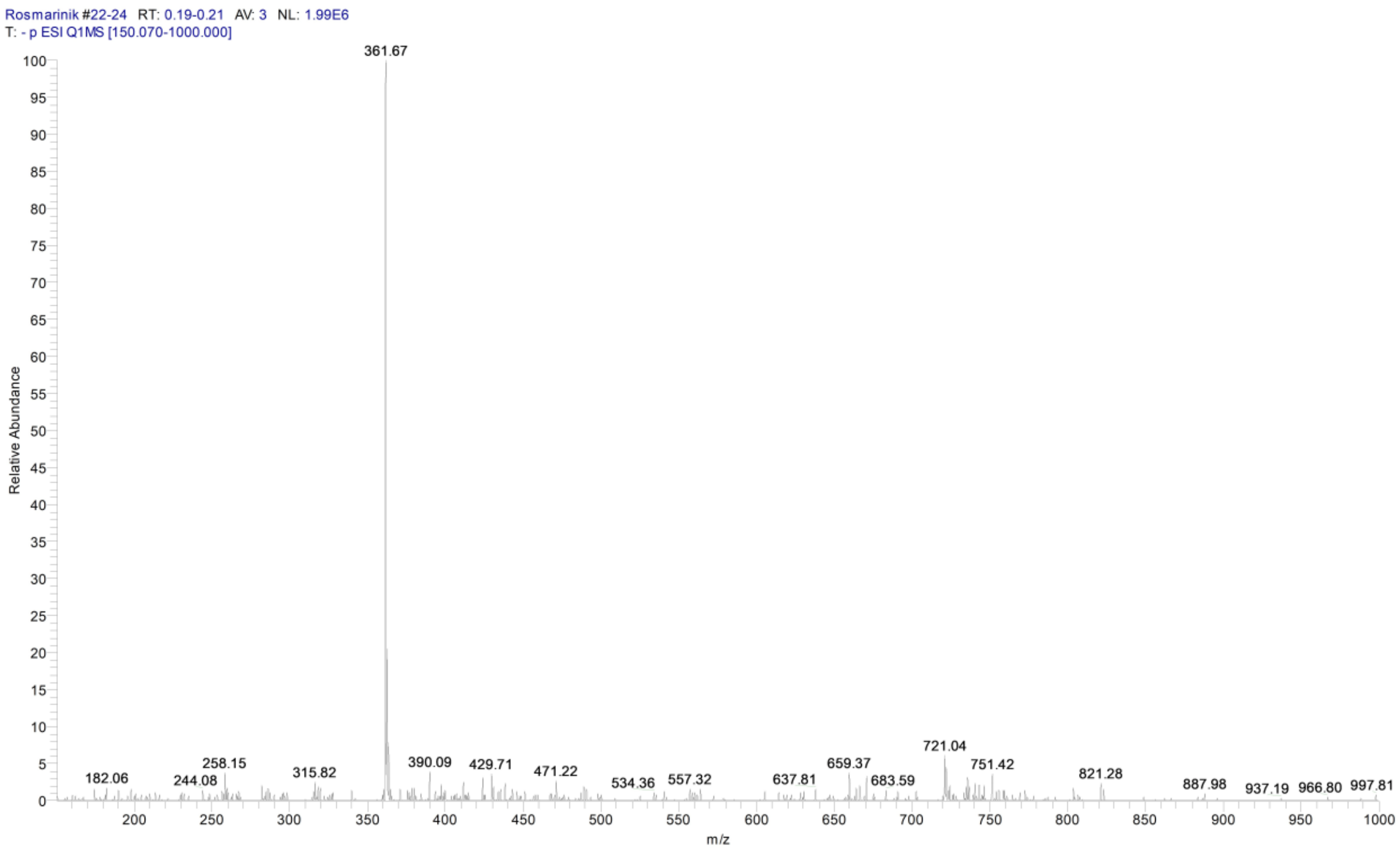

Figure S3. MS spectrum of RA.
